# mHealth Apps for Dementia Caregivers: Systematic Examination of Mobile Apps

**DOI:** 10.2196/58517

**Published:** 2024-11-20

**Authors:** Ning Zou, Bo Xie, Daqing He, Robin Hilsabeck, Alyssa Aguirre

**Affiliations:** 1 School of Computing and Information, University of Pittsburgh, Pittsburgh, PA, United States; 2 School of Nursing, The University of Texas at Austin, Austin, TX, United States; 3 School of Information, The University of Texas at Austin, Austin, TX, United States; 4 Glenn Biggs Institute for Alzheimer's & Neurodegenerative Diseases, Department of Neurology, University of Texas Health Sciences Center at San Antonio, San Antonio, TX, United States; 5 Department of Neurology, Dell Medical School, The University of Texas at Austin, Austin, TX, United States; 6 Steve Hicks School of Social Work, The University of Texas at Austin, Austin, TX, United States

**Keywords:** dementia, informal care, mobile health applications, tailoring, information quality, mobile phone

## Abstract

**Background:**

Informal caregivers of persons living with dementia are increasingly using mobile health (mHealth) apps to obtain care information. mHealth apps are seen as promising tools to better support caregivers’ complex and evolving information needs. Yet, little is known about the types and quality of dementia care information that these apps provide. Is this information for caregivers individually tailored; if so, how?

**Objective:**

We aim to address the aforementioned gaps in the literature by systematically examining the types and quality of care-related information provided in publicly available apps for caregivers of persons living with dementia as well as app features used to tailor information to caregivers’ information wants and situations.

**Methods:**

In September 2023, we used a multistage process to select mobile apps for caregivers of persons living with dementia. The final sample included 35 apps. We assessed (1) types of dementia care information provided in the apps, using our 3-item Alzheimer disease and related dementias daily care strategy framework, which encompasses educational information, tangible actions, and referral information; (2) quality of apps’ care information, using the 11 indicators recommended by the National Library of Medicine; and (3) types of tailoring to provide personalization, feedback, and content matching, which are common tailoring strategies described in the literature.

**Results:**

Educational information was the most prevalent type of information provided (29/35 apps, 83%), followed by information about tangible actions (18/35, 51%) and referrals (14/35, 40%). All apps presented their objectives clearly and avoided unrealistic or emotional claims. However, few provided information to explain whether the app’s content was generated or reviewed by experts (7/35, 20%) or how its content was selected (4/35, 11%). Further, 6 of the 35 (17%) apps implemented 1 type of tailoring; of them, 4 (11%) used content matching and the other 2 (6%) used personalization. No app used 2 types of tailoring; only 2 (6%) used all 3 types (the third is feedback).

**Conclusions:**

Existing dementia care apps do not provide sufficient high-quality, tailored information for informal caregivers. Caregivers should exercise caution when they use dementia care apps for informational support. Future research should focus on designing dementia care apps that incorporate quality-assured, transparency-enhanced, evidence-based artificial intelligence–enabled mHealth solutions for caregivers.

## Introduction

### Background

Alzheimer disease and related dementias (ADRD) present a critical public health concern; in 2023, a total of 55 million individuals were estimated to live with dementia worldwide, with an increase of nearly 10 million expected each year [1]. Often, the care for these persons living with dementia falls on *informal caregivers*—family members, friends, neighbors, or others (hereafter, “caregivers”). In the United States, more than 11 million caregivers were estimated to have provided approximately 18 billion hours of care to people living with dementia in 2022, valued at over US $330 billion [2].

Caregivers of persons living with dementia often lack formal training [2,3] or have little to no knowledge about dementia care [4], representing a substantial information gap [5,6], and they have health information wants (HIWs)—“health information that one would like to have and use to make important health decisions that may or may not be directly related to diagnosis or standard treatment” [7]. Centered around health consumer’s perspective, the ADRD HIWs framework suggests seven types of information that caregivers typically want for dementia care: (1) information about treatment or prevention, (2) characteristics of the patient’s health condition, (3) daily care strategies for patients at home, (4) practical information about care transition and coordination and end-of-life care, (5) psychosocial aspects of caregiving, (6) resources or scientific updates, and (7) legal, financial, or insurance-related information [8]. Unmet HIWs often lead to a severe care burden and increased stress for caregivers [9,10].

Given the anticipated increase in the number of persons living with dementia and the unlikelihood of a cure in the near future, numerous interventions have been developed to equip caregivers with necessary competencies to fulfill their HIWs (see review by Whitlatch and Orsulic-Jeras [11]). However, the feasibility of most currently available face-to-face interventions is limited due to the demanding nature of caregiving, challenges in finding alternative care for persons living with dementia to attend the programs, and the scarcity of caregiver services, especially in rural or underdeveloped areas [12]. Consequently, internet-based interventions such as web-based courses [13,14], or web-based programs [15,16] have emerged as a potentially efficient alternative, providing information and support to caregivers, as evidenced by the volume of systematic reviews and meta-analyses on this topic [17-19].

According to the review by Boots et al [17], internet-based interventions can be effective in supporting caregivers if they are tailored to the individual needs. For caregivers, timely and personally tailored information is important [20]. It improves their knowledge and enables them to develop coping abilities, so that they can provide competent care throughout the disease trajectory [21,22]. A recent meta-analysis of 17 randomized controlled trials of internet-based interventions has demonstrated improvements in dementia-related knowledge and care skills among caregivers [23]. Not surprisingly, it was discovered that personalized interventions are more beneficial than interventions that are not personalized. Despite these findings, a national caregiver survey in the United States reported that caregivers use health-related internet resources less frequently than the general public [24], which limits the reach and effectiveness of these programs for caregivers at large. Additionally, challenges such as the web layout’s inherent limitations and infrequent updates of features further hinder the delivery of tailored information [25,26], thus diminishing the potential effectiveness of internet-based interventions for caregivers. These findings underscore the need to explore more innovative technological solutions to better support caregivers’ complex and evolving HIWs.

### The Need of This Study

As of 2023, there has been a substantial increase in smartphone uptake among US adults aged 65 years or older, with adoption rates rising from 13% to 76% over the preceding decade [27,28]. Consequently, the use of mobile health (mHealth) apps to support caregivers has attracted much attention recently [29,30]. These internet-based applications, installed on mobile devices (eg, smartphones, wireless tablets), have become important tools for accessing health information and providing real-time feedback, even without computer and network connectivity [31]. They are now the most prevalent technological solutions [32] and have been used to support health education and the care across a wide range of chronic health conditions [33], and have also been tested among different age groups, including older adults [34].

Nevertheless, research on mHealth app–based interventions for caregivers is still at an early stage [29,35]. Recent literature reviews searched available apps, describing most apps as information resources for caregivers [36-38]. While these reviews acknowledged caregiving-related information as one of the most critical components of the apps, none of the studies systematically examined the quality of this information. Moreover, the extent to which these apps provide care-related information tailored to caregivers’ HIWs remains unclear. As Brodaty and Donkin [39] have noted, the success of caregiver interventions depends greatly on “the extent to which they are tailored to the needs of the individual and address issues to do with subjective burden.” Thus, it is critical to systematically examine mHealth apps for their information provision and tailoring, as a first step in assessing their effectiveness for the support of caregivers.

In the use of mHealth apps to tailor support [40,41], three strategies for tailoring are common: (1) *Personalization*—strategies that convey “explicitly or implicitly, that the communication is designed specifically” for an individual [42]. This is typically carried out by drawing on personally identifiable information, making clear that the messages are designed specifically for the individual, and situating the messages within the individual’s specific context. (2) *Feedback*—the provision of “messages to users about their psychological or behavioral states” that reflect user updates [41]. (3) *Content matching*, which entails providing content suitable for individuals’ stages of changes [40,42]. Reviews of mHealth apps for caregivers have not considered these strategies as components of tailoring [36,43]. In our study, we therefore systematically examine whether these tailoring strategies have been implemented in existing mHealth apps for caregivers of persons living with dementia, and if so, how they have been implemented.

### Context of This Study and Objectives

This study is an essential component of a larger project titled “Tailoring Responses to ADRD Caregivers' InfOrmation wants (TRACO) through Human-machine Collaboration” (R56AG075770). The primary goal of the project is to develop an artificial intelligence (AI)–based system with a mobile app interface that help caregivers in obtaining high-quality, relevant information tailored to their specific HIWs and unique caregiving contexts. While mHealth apps and AI tools can provide tailored information, their full tailoring potential has been underused [44-47]. Hence, this study is designed to systematically examine how mHealth apps provide information and serve as a foundational exploration to better support the development of the TRACO system.

Specifically, this study aims to understand more about the characteristics and the delivery of these apps. Therefore, the following research questions are identified:

1. What types of information do current mHealth apps offer to caregivers of persons living with dementia?
2. What is the quality of information presented in these mHealth apps?
3. How do current mHealth apps tailor their information to support caregivers of persons living with dementia?

## Methods

### Study Design

Following the PRISMA (Preferred Reporting Items for Systematic Reviews and Meta-Analyses) guidelines [48] and the procedures used in a previous systematic review of mHealth apps [49], we performed 3 rounds of screening to select relevant apps for examination. First, using keywords, we searched Apple’s App Store for iOS and Google’s Play Store for Android devices. Next, we screened the app store pages, using inclusion and exclusion criteria. Then we downloaded or attempted to download the apps to ensure their accessibility. Finally, using our criteria, we assessed the included apps in order to answer our research questions.

### Ethical Considerations

This study did not involve human participants, human data, or any form of intervention with individuals, and therefore did not require approval from an institutional review board or ethics committee. According to the Office for Human Research Protections, research that does not include human participants falls outside the scope of institutional review board review [50]. As no human participants were involved in the research, no ethical concerns related to human subject research were applicable.

### App Selection

Consistent with prior reviews [36,37,51], our search keywords included the following: “dementia care,” “dementia,” “Alzheimer’s care,” and “Alzheimer’s.” During initial screening, we realized that several apps’ titles used the abbreviation “Alz” instead of “dementia” or “Alzheimer’s”; we therefore expanded the search to include “Alz.” Our initial searches, performed in September 2023, yielded a total of 624 apps from Apple’s App Store for iOS and from Google’s Play Store for Android devices. We noted from the app store descriptions that many apps cater to various caregiver groups, not solely caregivers of persons living with dementia. These included caregivers of children with cognitive disorders or individuals with Ataxia, among others. Given our study’s specific aim to investigate mHealth apps exclusively for caregivers of persons living with dementia, we excluded apps not explicitly designed for dementia care at this stage. After removing duplicates, 127 apps remained.

We then screened the 127 apps for inclusion. Consistent with the scope of this study, we included only apps developed specifically to support caregivers. Apps that did not directly support the care of persons living with dementia were excluded. Specifically, we exclude (1) apps designed to support other dementia care stakeholders, such as health professionals, clinicians, or residential care facilities; (2) apps developed solely as cognitive screening and testing tools or brain exercise games, as they did not focus on caregiving for persons living with dementia. We also excluded (3) apps not in English to maintain consistency with the larger project as stated in the introduction, and (4) those that required the use of second device (eg, smartwatch or wearable gadget). The adoption of such devices typically involves interactions with multiple stakeholders, not just caregivers [52], which is beyond the scope of this study. Both free and paid apps were included.

The research team first discussed a randomly selected subset of 10 out of the 127 apps during weekly team meetings between September and October, 2023, using the established inclusion and exclusion criteria. Any disagreements were resolved through discussions. Subsequently, based on the agreed-upon criteria, 1 researcher (NZ) continued the screening process, ultimately including a total of 39 apps for further assessment. Then, 2 researchers (NZ and BX) independently reviewed a randomly selected sample of these 39 apps (n=13, representing 33% of the total). The agreement rate between the 2 reviewers was high (92.3%), and any differences were resolved through further discussions.

**Figure 1.**
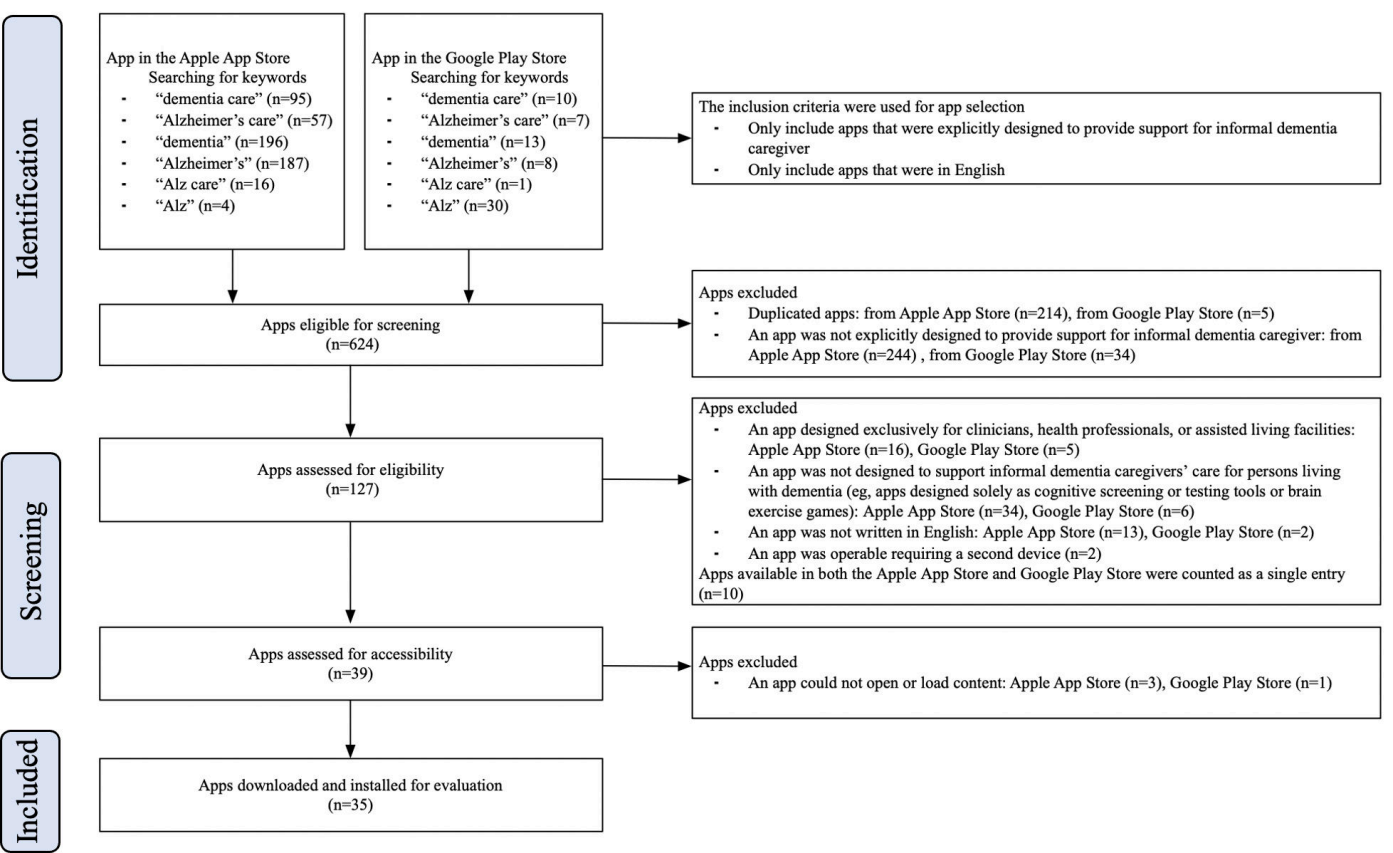
App selection process.

Finally, we downloaded or attempted to download each of the 39 apps to an iPhone and an Android phone for further examination. During this step, we excluded 4 more apps that could not be opened or downloaded, for a final sample of 35 apps: 20 were iOS-only; 5 were Android-only; and 10 were available for both iOS and Android systems. App selection, including the numbers of apps excluded at each step, is illustrated in Figure 1.

### Measures

#### The ADRD Daily Care Strategy Framework to Evaluate Types of Information Provided

To assess the types of dementia care information provided in the apps, we used the ADRD Daily Care Strategy Framework developed in our earlier work [53]. We selected this framework because the types of information it encompasses have been proven in our prior work to be evidence-based care strategies that effectively match the responses to caregivers’ HIWs. This framework includes 3 types of information about care strategies: educational information, tangible actions that caregivers can take; and referrals for caregivers to seek help beyond the patient-caregiver dyad (Table 1). Each type of information found in the app was assigned a score of 1, and its absence was scored as 0; scores thus ranged from 0 to 3. Higher scores indicated more types of care information present in the apps.

**Table 1.** The Alzheimer disease and related dementias daily care strategy framework.

Types of information provided	Operational definition
Educational information	Provides education about the behavior or situation in question; education about what caregivers could do on their own in response to challenges encountered; focus on caregivers’ own knowledge, preparation, and adjustments, not on others’
Tangible actions	Provides information about specific things caregivers can do (on their own) to address situations
Referral information	Provides referral information so that caregivers can seek help beyond the patient-caregiver dyad, for example, peer caregivers, health care professionals, financial advisors, an attorney for elder law, other family members, community organizations, and long-term care facilities

#### The National Library of Medicine’s Criteria for Evaluating the Quality of Web-Based Health Information

The National Library of Medicine (NLM)’s tutorial on evaluating web-based health information offers a comprehensive checklist for assessing the quality of web-based health information [54].

Although the Mobile App Rating Scale [55] is commonly used to evaluate mHealth apps for caregivers [38,51], its criteria for assessing the quality of information—ensuring the app content is correct, well-written, and relevant to the goal or topic of the app—appear too vague for comprehensively evaluating the care-related information provided by the apps. Drawing on the success of previous empirical research that used the NLM’s criteria to assess information quality in mHealth apps for cardiovascular disease [49], we adopted the NLM criteria and developed definitions for the context of dementia care to operationalize the NLM’s criteria (Table 2). These included 11 indicators of information quality; the presence of each indicator was recorded as 1, and each absence was recorded as 0 (scoring range: 0‐11). Higher scores indicated higher information quality.

**Table 2.** National Library of Medicine’s criteria for information quality and our operational definitions.

Evaluation criteria	Operational definition
Providing information on who is managing the app	Provides information that could help users understand who oversees the app (eg, information about the app provider’s name). Such information is typically found via the *About Us* button in the app or on the app’s store page.
Providing information about why the app is being provided	Provides information that could help users understand the app’s purpose, intended users, and functionalities. Such information is typically found on the app’s store page or via the *About this app* or *About us* button in the app (eg, indicating that the app is developed for dementia caregivers, or to provide dementia care-related information, or to provide tailored support to dementia caregivers).
Providing the app provider’s contact information	Provides information about the physical address, a contact number or email address for the app developer or administrator, or an option for users to submit questions or comments.
Providing information on the source of the content	Provides information that could help users understand where the dementia care information used by the app comes from (eg, an article or book with authors’ names; or for web-based information, the website from which the information was retrieved).
Providing information on how the content was selected	Provides a logical explanation for how the app’s information was selected (eg, information selected from peer-reviewed journals).
The content that goes on the app is reviewed by an expert	Provides information to make clear that information presented in the app has been reviewed by qualified health care professionals.
Does not use unbelievable or emotional claims	Whether or not the app makes claims that are too good to be true or are based on emotions instead of scientific facts.
Content is up to date	The original National Library of Medicine guidelines do not specify what time frame would be considered up to date; in our study, we operationalized this indicator as app content updated in the past 12 months.
Financial disclosure	Provides information on where the money to support an app comes from (eg, government agencies, nonprofit organizations, or professional service companies). This information can help users understand whether an app’s providers have financial motives that users should be aware of (eg, the sale of courses or consultation services).
Does not have advertisements	Whether or not an app contains advertisements. Note: if a treatment option mentioned in an app was a part of scientific results (eg, reported in a research article), then it was not considered an advertisement.
Personal data privacy disclosure	Whether or not an app asks users to submit personal information (eg, name, phone number, or email address) and personal health indicators (eg, health status or medical history) in order to use certain app features. If asked, whether an app provides information on how users’ personal data will be used.

#### Types of Tailoring Strategies for Evaluating Apps’ Information Tailoring

We examined whether apps provided the 3 types of tailoring strategies commonly reported in the literature: personalization, feedback, and content matching [40,42]. Our operational definitions for these 3 types of tailoring are provided in Table 3. The presence of each type of tailoring was recorded as 1, and each absence was recorded as 0 (scoring range: 0‐3). Higher scores indicate more types of tailoring.

**Table 3.** Information tailoring strategies and our operational definitions.

Evaluation criteria	Operational definitions
Personalization	App’s output contains personal information (based on the user’s input, for example, the person living with dementia is the user’s grandmother) to make clear that the output was designed specifically for the user and places the messages in the user’s specific context.
Feedback	App’s output contains information acknowledging the user’s specific situation (based on the user’s input) to convey to the user that the system is aware of the user’s situation.
Content matching	App’s output contains relevant responses to the types of health information wants expressed in a user’s input.

### Data Analysis

We assessed (1) types of dementia care information provided in the apps, using our 3-item ADRD Daily Care Strategy Framework; (2) quality of dementia care information provided in the apps, using the 11 indicators recommended by the NLM; and (3) types of tailoring provided, using the 3 common tailoring strategies reported in the literature. Further, 2 members of the research team (NZ and BX) initially evaluated 7 randomly selected apps from the final sample according to these measures. For any items with a recorded disagreement, the 2 reviewers met to discuss and reach a consensus. Later, researcher NZ continued and completed the evaluation of the remaining apps. Discussions were consistently held between researchers NZ and BX to address any ambiguities that arose during the evaluation process. The ratings for each measure were entered into a Microsoft Excel spreadsheet, and we used descriptive statistics to analyze each rating.

## Results

### Overview

Of the 35 apps in our final sample, 10 (29%) were developed between 2012 and 2017. Among them, only 1 (3%) was still having regular updates at the time of our study. Further, 6 (17%) of the apps had actively received updates, for an average duration of 5 (SD 1.55) years before stopping any further updates; 3 had not been updated at all since their initial release. The other 25 (71%) apps were developed between 2018 and 2023. Additionally, 13 (37%) were actively receiving updates by 2023; 7 (20%) had not received any updates since their release; and another 5 (14%) stopped receiving updates after an average of 2.4 (SD 1.95) years.

In total, 15 (43%) apps were created by for-profit companies such as corporations providing health care services or consulting firms; 8 (23%) were developed by nonprofit health care organizations or charities; another 8 (23%) were developed by academic institutions; and the remaining 4 (11%) did not disclose any affiliation of app developers. Most apps were free (28/35; 80%); 5 (14%) were free with in-app purchases; only 2 (6%) were paid apps (requiring US $2.99 and US $3.99, respectively). The largest number of apps (15/35, 43%) were from the United States; 8 (23%) from the United Kingdom; 4 (11%) from Australia, 3 (9%) from Canada, and 1 (3%) from India. The 4 (11%) apps that provided no developer information also provided no information about where they were developed.

### Types of Information

In total, 16 (46%) of the apps offered only 1 type of information. Among them, 12 (75%) provided educational information; 2 (13%) offered information about tangible actions, and 2 (13%) offered referral information. Further, 12 (34%) apps offered 2 types of information: 7 (58%) of them offered both educational information and information about tangible actions; 3 (25%) provided both educational information and referral information; and 2 (13%) provided both information about tangible actions and referral information. Only 7 (20%) apps in our final sample offered all 3 types of information. Of the reviewed apps, if an app attempted to offer more than one type of information, its content frequently remained broad and merely basic, in contrast to apps that specialized in providing only a specific type of information. Overall, educational information was the most commonly offered type of information (29/35, 83%), followed by information about tangible actions (18/35, 51%). Information about referrals (14/35, 40%) was the least common type of information provided in the apps.

### Information Quality

Of the 35 apps, 17% (6 apps) met 3‐4 of the NLM’s criteria for web-based health information quality. Another 34% (12 apps) met 5‐6 criteria, and 40% (14 apps) met 7‐9 criteria. Only 11% (4 apps) met 10 of the NLM’s criteria, and none met all 11 criteria. Notably, the 4 apps that met 10 criteria were all developed by academic institutions, nonprofit health care organizations, or charities. In contrast, all 6 apps meeting only 3 or 4 criteria were developed by for-profit companies or developers of unknown origin. Apps available on both iOS and Android systems scored higher on average (mean 7.9, SD 2.28, 95% CI 7.14-8.66) than those available exclusively on iOS (mean 6.6, SD 1.73, 95% CI 6.03-7.17) or Android (mean 4, SD 1.41, 95% CI 3.53-4.47). All apps met 2 of the NLM criteria: they clearly stated the purpose of developing the app and avoided making unbelievable or emotional claims. Few explained whether the app’s content was reviewed by experts (7/35, 20%); and even fewer stated how the content was selected (4/35, 11%; Table 4).

**Table 4.** National Library of Medicine’s information quality indicators covered by the apps.

Information quality indicator	Apps, n (%)
Provided information about why the app is being provided	35 (100)
Does not use unbelievable or emotional claims	35 (100)
Provided information on who is managing the app	30 (86)
Does not have advertisements	29 (83)
Provided the app provider’s contact information	25 (71)
Personal information use disclosure	22 (63)
Provided information on the source of the content	18 (51)
Content is up to date	15 (43)
Financial disclosure	13 (37)
The content that goes on the app is reviewed by an expert	7 (20)
Providing information on how the content was selected	4 (11)

### Information Tailoring

In total, 6 of the 35 (17%) apps implemented 1 type of tailoring strategy; of them, 4 (11%) used content matching and the remaining 2 (6%) used personalization. The specific content matching strategies varied among the 4 apps. Further, 2 of the apps used web-based discussion forums to deliver responses from platform-verified experts or peer caregivers. The other 2 offered a list of behavioral problems along with possible causes, allowing caregivers to choose, and, on the basis of the caregivers’ choice, the app tailored a list of care strategies to address the specific problem expressed by the caregiver.

Of the 2 apps that entailed personalization, each used a unique approach to collect and use personal information. However, neither app successfully delivered tailored support. The first app gathered detailed information on the person living with dementia, such as disease stage and health conditions. The second app surveyed caregivers’ stress levels and the person living with dementia’s behavioral issues. Despite collecting such personal information, both apps failed to tailor the content depending on user input and provided the same responses regardless of the user data provided. This lack of customization and transparency about how personal information affected content raises concerns about the apps’ effectiveness and credibility for offering personalized support.

No app used 2 types of tailoring strategies; 2 (6%) used all 3 tailoring strategies. Both apps used generative AI-enabled intelligent assistants to provide tailored responses. These platforms allowed caregivers to input any queries and provided personalized answers. One of these apps explicitly stated that it used ChatGPT for this purpose; the other did not specify what AI tool it used or how its intelligent assistant was developed.

## Discussion

### Principal Findings

Previous reviews have found that dementia care apps mainly offer educational content along with generic care tips for coping [36,37]. In our study, we have examined the extent to which care-related information in the apps includes not only educational information but also information about tangible actions and referrals. Tangible action and referral information were underrepresented in the apps. As these 2 types of information can vary greatly depending on caregivers’ specific situations [56,57], it may be challenging for apps to provide such information. Current dementia care apps primarily provide extensive static educational information, and so they cannot meet caregivers’ specific HIWs [36,38]. Future dementia care apps should consider including elements designed to capture caregivers’ specific HIWs, which can evolve as the disease progresses [8].

Recent reviews have primarily used the Mobile App Rating Scale [55] to evaluate apps and have found that the quality of information in dementia care apps falls below minimum acceptable scores [38,51]. Going beyond statistical validation of this known issue regarding poor information quality [36], we have adapted the NLM’s information quality evaluation criteria to examine specific attributes that affect information quality. Apps developed by academic institutions or nonprofit health care organizations appeared to align more closely with the NLM’s quality indicators. Nevertheless, the lack of indicators for expert-reviewed content and content selection remains a problem. Given the critical role of health care providers in ensuring the accuracy and reliability of information [49,58], further research is needed to explore how to design features that integrate dementia care professionals within information assessment and selection [58,59]. Implementing these improvements will enhance the credibility and reliability of caring-related information and potentially improve caregivers’ information-seeking and, ultimately, the apps’ adoption rates [60].

Commonly recommended tailoring has yet to be successfully implemented in dementia care apps. By and large, apps’ tailoring merely replicates approaches found in web-based discussion forums [61,62]. Yet, there is potential for dementia care apps to provide more tailored, personally relevant learning [36]. The AI-enabled intelligent assistants found in 2 of the reviewed apps may represent a promising approach for delivering tailored information. Recent research suggests ChatGPT’s capability to generate high-quality responses to meet dementia caregivers’ HIWs [63]. Nonetheless, recent studies have raised concerns regarding ChatGPT’s use in health care, particularly its tendency to conceal its information sources and provide inappropriate references [64]; its outputs may require cautious human oversight to ensure quality [65].

These concerns echo our findings for apps’ lack of explanation regarding information selection, including a lack of information about whether their content had been reviewed by health care professionals. If unaddressed, these issues are likely to persist in AI-enabled mHealth apps for caregivers. More rigorous efforts are needed in dementia care research and practice to investigate AI-enabled mHealth tools. There is a need to explore comprehensive frameworks that involve health care professionals in content validation, as well as advancements to streamline these technological processes.

### Limitations and Future Directions

Limitations of this study include the following. App evaluation was carried out by researchers with professional expertise in the subject matter; no evaluation was performed by caregivers. Our evaluation relied on guidelines designed from a top-down perspective, reflecting what health care professionals would view as important for evaluating an app’s information quality. This approach might not fully align with caregivers’ perspectives, and the insights derived might differ from those of caregivers. Given the importance of user-centered design in developing mHealth technology for dementia caregiving [66], future research will benefit from caregivers’ evaluation of such technology. User-centered evaluation would ensure that apps meet caregivers’ actual needs and preferences. This study only included mHealth apps that were available in English, thus, there could be apps available in other languages that provide high-quality information to caregivers of persons living with dementia. This study did not consider app design or usability when evaluating the type and quality of information provided. Given that academic institutions and nonprofits produced mHealth apps with higher information quality, future developments could include collaborations with for-profit organizations to design tailored apps that meet the needs of caregivers of persons living with dementia.

### Conclusion

Tailoring and the quality of care information is limited in current mHealth apps for caregivers. Caregivers should use current dementia care apps with caution when they seek information about caregiving and support. Although mHealth may potentially be effective in meeting caregivers’ HIWs, future research is needed in order to develop quality-assured, transparency-enhanced, evidence-based AI-enabled mHealth solutions for caregivers.

## Abbreviations

ADRD: Alzheimer disease and related dementias
AI: artificial intelligence
HIW: health information want
mHealth: mobile health
NLM: National Library of Medicine
PRISMA: Preferred Reporting Items for Systematic Reviews and Meta-Analyses
TRACO: Tailoring Responses to Alzheimer Disease and Related Dementias Caregivers' Information
